# Elucidating the Molecular Network Underpinning Hypoxia Adaptation in the Liver of Silver Carp (*Hypophthalmichthys molitrix*) via Transcriptome Analysis

**DOI:** 10.3390/ani15243577

**Published:** 2025-12-12

**Authors:** Xiaohui Li, Long Ding, Nannan Feng, Hang Sha, Guiwei Zou, Hongwei Liang

**Affiliations:** 1 Yangtze River Fisheries Research Institute, Chinese Academy of Fishery Sciences, Wuhan 430223, China; lixiaohui@yfi.ac.cn (X.L.); d1911651249@163.com (L.D.); fengnannan@yfi.ac.cn (N.F.); sh1812@yfi.ac.cn (H.S.); zougw@yfi.ac.cn (G.Z.); 2 College of Animal Science, Anhui Science and Technology University, Chuzhou 233100, China; 3 Key Laboratory of Aquatic Genomics, Ministry of Agriculture and Rural Affairs, Chinese Academy of Fishery Sciences, Beijing 100141, China

**Keywords:** *Hypophthalmichthys molitrix*, hypoxia adaptation, liver, transcriptome

## Abstract

The fish liver serves as a crucial metabolic organ, integral to detoxification, nutrient storage, and energy regulation, thereby playing a pivotal role in enabling organisms to adapt to environmental fluctuations. The silver carp (*Hypophthalmichthys molitrix*), an important species in Chinese freshwater aquaculture, demonstrates limited tolerance to hypoxic conditions. Nevertheless, the alterations in gene expression patterns within the liver of silver carp under hypoxic stress are not yet fully elucidated. In this study, we exposed silver carp to hypoxic conditions using a natural oxygen depletion method and utilized RNA sequencing to investigate transcriptional regulation in the liver across varying levels of hypoxic stress. We identified a total of 628 differentially expressed genes (DEGs), with 42 being common across all stress conditions. These DEGs were classified into four groups based on their expression trends and subjected to GO enrichment analysis, which revealed significant enrichment in terms associated with the endoplasmic reticulum, cell proliferation, myofibrils, and sterol metabolic processes. The KEGG enrichment analysis identified nine pathways that were consistently and significantly enriched across all stress levels, six of which encompass the maintenance of cellular homeostasis, metabolic regulation, and immune modulation. The elucidation of the molecular network associated with hypoxia adaptation in the liver of silver carp presented in this study provides crucial theoretical insights into the mechanisms underlying hypoxia tolerance in fish.

## Simple Summary

The silver carp (*Hypophthalmichthys molitrix*) demonstrates a pronounced sensitivity to hypoxic conditions, which presents considerable challenges to both its survival and the sustainable advancement of its aquaculture industry. Presently, there is an insufficient systematic comprehension of the molecular response mechanisms employed by silver carp when subjected to hypoxic stress. This study investigates alterations in liver gene expression and associated biological processes in silver carp exposed to varying levels of hypoxic stress, with the objective of systematically elucidating the molecular regulatory mechanisms that underpin their adaptation to hypoxia. The results will offer a crucial theoretical foundation for enhancing the understanding of the physiological adaptation strategies employed by silver carp in response to hypoxic environments.

## 1. Introduction

Dissolved oxygen (DO) is a key indicator for assessing water quality in aquaculture ecosystems. Its level directly affects fish respiration and significantly influences physiological processes such as swimming behavior, growth, and metabolism [1]. However, under the combined influence of natural disturbances like global warming [2] and anthropogenic disturbances such as water pollution [3], algal blooms [4], and high-density farming [5], aquatic ecosystems are continuously stressed. This leads to frequent localized hypoxia, posing a severe threat to the survival of aquatic organisms. Such hypoxic conditions present significant challenges to cultured species, frequently leading to significant declines in growth performance and survival rates [6].

Typically, hypoxia is defined as occurring when the dissolved oxygen concentration in water falls below 2.0 mg/L [7]. Under these conditions, excessively low DO levels severely disrupt cellular energy metabolism, inhibit normal ATP synthesis, and cause excessive accumulation of reactive oxygen species (ROS). This triggers oxidative damage and dysfunction at the cellular level [8]. Prolonged hypoxia also impairs fundamental fish metabolism (such as interfering with protein synthesis and nitrogen metabolism), ultimately leading to adverse outcomes like growth retardation [9].

In freshwater aquaculture, the silver carp is highly valued for its significant economic and ecological importance. This species is highly favored by aquaculturists due to its rapid growth rate, strong disease resistance, and high yield [10]. As a filter-feeding fish, silver carp play a crucial role in controlling algal blooms by consuming plankton, thereby improving water quality and maintaining the ecological balance of aquaculture systems [11,12]. However, silver carp are highly sensitive to environmental changes and are particularly prone to stress responses under hypoxic conditions, leading to aquaculture losses that threaten the stability and sustainability of the industry [13].

The liver is a vital metabolic organ in fish and serves as a central hub for adaptive regulation. Research indicates that the liver establishes a complex adaptive network by integrating multiple physiological functions, including metabolic reprogramming, detoxification, immune response modulation, activation of antioxidant defense systems, and maintenance of hormonal balance [14]. This multidimensional regulatory mechanism not only optimizes energy metabolism efficiency but also significantly enhances the organism’s capacity to adapt to environmental changes, enabling fish to effectively counteract physiological stress induced by adverse factors such as hypoxia. Nevertheless, the specific physiological responses and adaptive mechanisms of the silver carp liver to hypoxia stress remain incompletely understood.

Transcriptome sequencing technology has significantly accelerated biological and genetic research by enabling the identification of gene expression differences and their correlation with specific traits, thereby uncovering complex regulatory networks [15]. Recent years, transcriptome analysis has been widely employed to identify differentially expressed genes in various aquatic species, including bighead carp (*Hypophthalmichthys nobilis*), Chinese mitten crab (*Eriocheir sinensis*), and darkbarbel catfish (*Pelteobagrus vachelli*) under hypoxic conditions [16,17,18], furthermore to analyze the response patterns. However, limited research has been conducted on transcriptome analysis of silver carp liver under hypoxic treatments.

In this study, the transcriptome of silver carp liver was comparatively analyzed under different hypoxia levels using RNA-seq technology. The results provide novel insights into the adaptation mechanisms of silver carp to hypoxic stress and offer a theoretical foundation for exploring its physiological functions and molecular activity patterns under hypoxic conditions.

## 2. Materials and Methods

### 2.1. Experimental Fish and Hypoxia Treatment

The silver carp used in this study were obtained from the Yaowan Experimental Station of the Yangtze River Fisheries Research Institute, Chinese Academy of Fishery Sciences (Jingzhou, Hubei Province, China).

Before initiating the experiment, 120 healthy fish, with a mean body weight of 186.36 ± 8.52 g and an average body length of 20.44 ± 0.75 cm, underwent a two-week acclimation period in tanks equipped with a recirculating freshwater system. During this period, the water temperature was consistently maintained at 23.0 ± 0.5 °C, and dissolved oxygen levels exceeded 6.0 mg/L. Additionally, the fish were fasted for 24 h prior to the experiment. At the onset of the experiment, 12 glass water tanks were utilized. Three tanks (*n* = 10) were maintained under normoxic conditions as control group, while the remaining nine tanks (*n* = 10) served as the experimental groups. Three tanks per group, serving as three replicates. Hypoxia experiments were conducted by sealing the water-filled tanks with plastic film, allowing the fish to gradually deplete the oxygen in the water through natural respiration. This process continued until the majority of the fish exhibited mouth-breathing behavior (three tanks, designated as the hypoxia group, with dissolved oxygen levels of 0.75 ± 0.05 mg/L, approximately half of the fish lost equilibrium (three tanks, designated as the semi-asphyxia group, with dissolved oxygen levels of 0.60 ± 0.04 mg/L, and approximately all the fish experienced a loss of balance due to the inability of their gill lamellae to open and close rhythmically, resulting in sinking (three tanks, designated as the asphyxia group, with dissolved oxygen levels of 0.27 ± 0.03 mg/L [13,19] (Table S1). In this study, water temperature, pH, and DO were monitored continuously using a HACH portable multiparameter meter (HACH, HQ40d, Loveland, CO, USA).

### 2.2. Sample Collection

Anesthesia was administered to fish from all four groups using 100 ppm tricaine methanesulfonate (MS-222; Sigma-Aldrich, St. Louis, MO, USA), after which liver tissue samples were obtained from ten fish in each tank. All samples were quickly frozen in liquid nitrogen and maintained at −80 °C for future analysis. The experimental process in this study was approved by the Animal Ethics Committee of the Yangtze River Fisheries Research Institute, Chinese Academy of Fisheries Sciences (protocol code: 2020-LXH-01).

### 2.3. Determination of the Oxidative Stress Indices

Approximately 0.5 g of frozen liver tissue was weighed accurately, and 9 volumes of pre-cooled normal saline (0.86%, *w*/*v*) were added at a weight-to-volume ratio of 1:9. A 10% tissue homogenate was prepared using an automatic tissue homogenizer. Subsequently, the homogenate was centrifuged at 2500 r/min for 10 min under ice-water bath conditions. The resulting supernatant was collected for the determination of superoxide dismutase (SOD), catalase (CAT), and glutathione peroxidase (GPX) activities, with all samples required to be assayed within 12 h. All enzyme activity measurements were performed strictly following the instructions provided with the commercial assay kits purchased from Nanjing Jiancheng Bioengineering Institute.

### 2.4. RNA Extraction and Sequencing

To reduce inter-individual variability, liver tissues from five randomly selected fish per replicate were pooled for RNA extraction. Total RNA was extracted from both control and experimental samples using TRIzol reagent (Invitrogen, Waltham, MA, USA). The cDNA library was prepared and sequenced by Wuhan Fraser Genetic Information Co. (Wuhan, China) using the Illumina HiSeq^TM^ 2500 system (Illumina, San Diego, CA, USA), which generated 150 bp paired-end reads. All the data are available at the NCBI SRA database (Accession numbers: SRR18863890–SRR18863901).

### 2.5. Transcriptome Data Analysis

Quality control of the FASTQ output files was conducted using FASTQC (version 0.11.5). Adapter sequences were subsequently removed using Trimgalore (version 0.4.3). The processed reads were aligned to the reference genome of silver carp (GCA_041475455.1). Raw counts were generated using HTSeq (version 0.6.1) [20]. DESeq2 (version 1.30.1) was used to conduct differential expression analysis, incorporating sequential pairwise condition tests and likelihood ratio tests (LRT) [21]. Differentially expressed genes were identified based on a fold change (FC) greater than 2.0 and a *p*-adjusted value less than 0.05 (FDR) [22]. GO and KEGG enrichment analysis of DEGs was performed using the OmicStudio tools (provided by Gene Denovo Biotechnology Co., Guangzhou, China).

### 2.6. Real-Time Quantitative PCR (RT-qPCR) Validation

Total RNA was reversed transcribed using the EasyScript^®^ One-Step gDNA Removal and cDNA Synthesis SuperMix (TransGen, Beijing, China) according the protocol. The cDNA was subjected to quantitative real-time PCR (RT-qPCR) analysis on an ABI 7500 Real-Time PCR System (Applied Biosystems, San Francisco, CA, USA) with gene-specific primers. Given its stable expression across all tissues of silver carp, 40S ribosomal RNA was employed as an internal reference. The primer sequences are provided in Table S2. Gene expression levels were quantified using the comparative Ct method (2^−ΔΔCt^) [23], with calculations performed using QuantStudio™ real-time PCR software (version 1.2).

### 2.7. Statistical Analysis

Statistical analysis was performed using GraphPad Prism software (version 8.0). All experimental measurements were repeated at least three times, data were expressed as mean ± standard deviation. One-way analysis of variance (ANOVA) followed by multiple comparison tests was applied to confirm the statistical significance of differences among groups. Pearsons’s correlation analysis was employed to assess the consistency between the RT-qPCR data and RNA-Seq data.

## 3. Results

### 3.1. The Changes in Oxidative Stress Indices After Hypoxia

**Figure 1:**
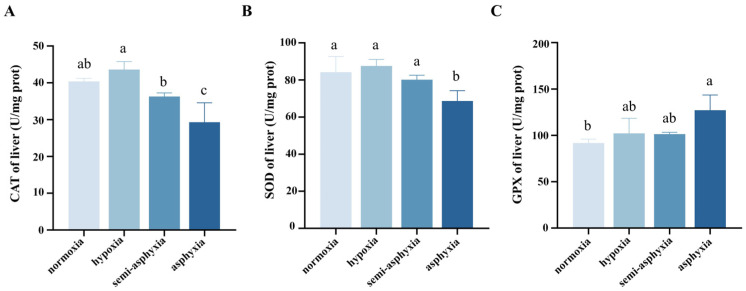
Changes in (**A**) catalase (CAT), (**B**) superoxide dismutase (SOD), and (**C**) glutathione peroxidase (GPX) in the liver tissue of silver carp under hypoxic stress. Different lowercase letters in the superscript indicate significant differences (*p* < 0.05), while the same letter indicates no significant difference (*p* > 0.05).

As dissolved oxygen levels declined, the activities of catalase (CAT) and superoxide dismutase (SOD) in the liver of silver carp showed an initial increase followed by a decrease, whereas glutathione peroxidase (GPX) activity exhibited a continuous upward trend throughout hypoxia exposure. Specifically, in the hypoxic group, CAT activity rose to its highest level but did not differ significantly from the normoxic group. As oxygen decreased further, CAT activity declined steadily, reaching its lowest point in the asphyxia group (*p* < 0.05; Figure 1A). SOD activity peaked in the hypoxic group during hypoxia stress then continued to decrease, with the asphyxia group showing significantly lower activity (*p* < 0.05; Figure 1B). In contrast, GPX activity increased progressively under hypoxia stress and reached its maximum in the asphyxia group (*p* < 0.05; Figure 1C).

### 3.2. Mapping of Reads Generated by RNA-Seq of Liver to the Silver Carp Genome

To examine the liver’s transcriptional response to hypoxic stress, we extracted total RNA and conducted RNA-seq analysis on three biological replicates across four experimental conditions: normoxia, hypoxia, semi-asphyxia, and asphyxia. This resulted in the construction of 12 cDNA libraries. A total of 320,850,557 high-quality clean reads were obtained, with 247,597,465 reads successfully mapped to the silver carp genome (Table 1). The average mapping rate of 78.04% suggests that the sequencing data were of high quality. Although the mapping rate of one sample (semi-asphyxia_3) was slightly lower, the number of detected expressed genes showed no difference compared to the other samples (Table S3). The PCA diagram indicated repeatability and heterogeneity of sequencing samples (Figure S1).

### 3.3. Differentially Expressed Genes in Liver Under Hypoxia Stress

**Figure 2:**
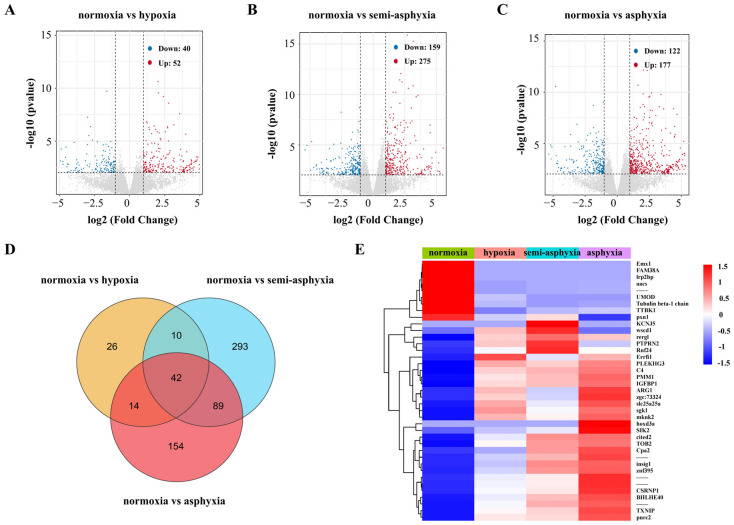
Analysis of DEGs in the liver tissue of silver carp under varying degrees of hypoxia stress. (**A**–**C**) Volcano plots illustrating DEGs from the three comparative groups. Red dots denote upregulated genes, while blue dots indicate downregulated genes, grey dots indicate no differential genes. (**D**) Venn diagram showing the distribution of DEGs across the three comparisons. (**E**) Heatmap generated from FPKM values, illustrating expression patterns of overlapping DEGs. Genes with expression levels above the mean are highlighted in red, and those below the mean are shown in blue.

The read counts of genes were adjusted to FPKM values, representing fragments per kilobase of transcript per million mapped reads, for each sample (Table S4) and subsequently analyzed for differential gene expression. Compared to the normoxic group, the hypoxic group exhibited 92 DEGs, with 52 genes up-regulated and 40 genes down-regulated (Figure 2A, Table S5). In the semi-asphyxia group, 434 DEGs were identified, comprising 275 up-regulated and 159 down-regulated genes (Figure 2B, Table S4). The asphyxia group revealed 299 DEGs, consisting of 177 up-regulated and 122 down-regulated genes (Figure 2C, Table S5). Venn diagram analysis indicated that 42 DEGs were commonly expressed across all three treatment groups (hypoxia, semi-asphyxia, and asphyxia) (Figure 2D, Table S6). These 42 shared DEGs were subsequently visualized through heatmap analysis (Figure 2E, Table S7).

### 3.4. GO Enrichment Analysis of DEGs

**Figure 3:**
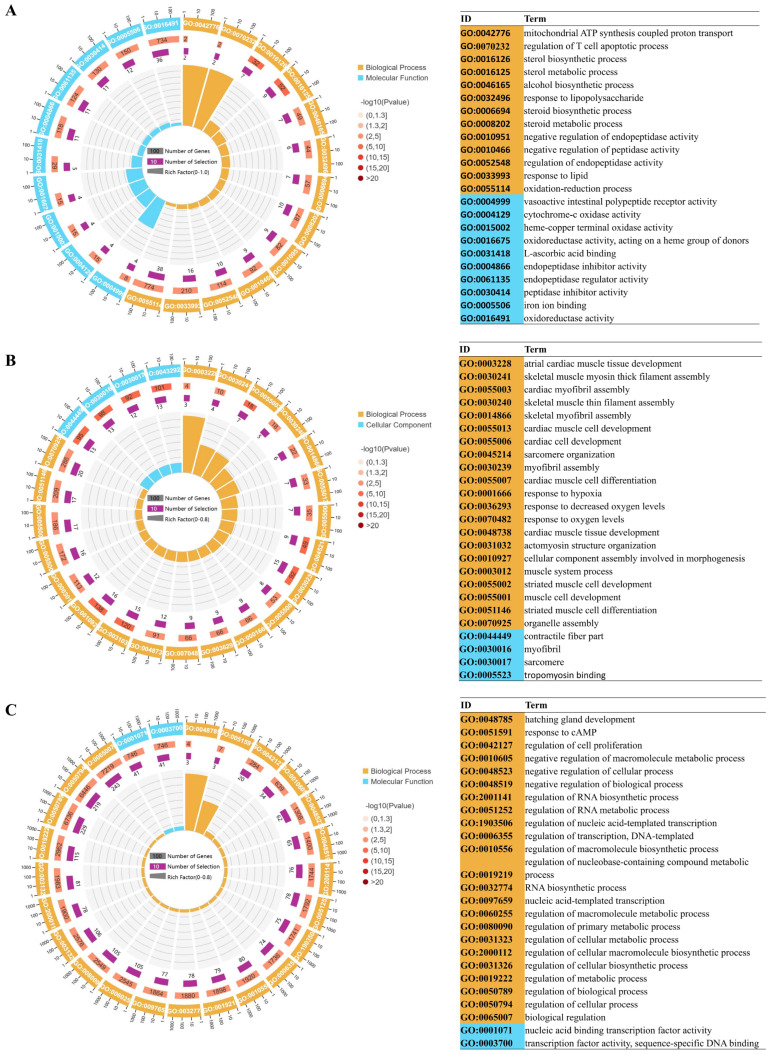
The 25 most significant terms identified from the GO enrichment analysis were demonstrated with cycle graph. (**A**) normoxia vs. hypoxia, (**B**) normoxia vs. semi-asphyxia, (**C**) normoxia vs. asphyxia. The innermost circle illustrates the enrichment factor, while the second circle depicts the quantity of upregulated and downregulated input genes. The third circle signifies the total number of background genes associated with the GO terms. The outermost circle, annotated with scales indicating gene numbers, corresponds to the GO term identifiers. Accompanying this visual representation, a table on the right offers comprehensive details regarding the GO terms.

A systematic characterization of the functional classification and biological properties of DEGs were provided through GO annotation analysis (Figure 3 and Table S8). DEGs from the three comparison groups were annotated using GO analysis. Taking the normoxia vs. hypoxia comparison as an example, the principal categories identified were as follows: within the biological process category, the predominant subcategories included “sterol metabolic processes” (GO:0016125), “sterol biosynthetic processes” (GO:0016126), and “redox processes” (GO:0055114). In the molecular function category, the primary subcategories were “vasoactive intestinal peptide receptor activity” (GO:0004999), “oxidoreductase activity” (GO:0016491), “endopeptidase inhibitor activity” (GO:0004866), and “endopeptidase modulator activity” (GO:0061135).

**Figure 4:**
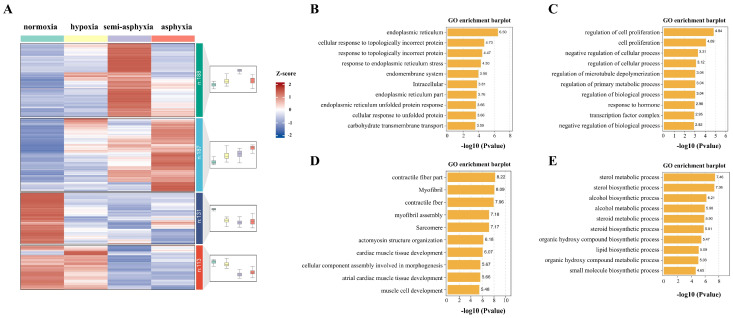
Heatmaps were utilized for the analysis of expression trends of all DEGs and to elucidate the functional characteristics of genes within each cluster. The ten most significantly enriched GO terms were identified for the first, second, third, and fourth clusters, respectively, as depicted in Figures (**A**–**E**). Blue denotes low expression levels, whereas red signifies high expression levels.

To gain deeper insights into the biological functions of the DEGs, those exhibiting similar expression patterns were systematically clustered. Consequently, a total of 628 DEGs identified across the three groups were classified into four distinct clusters, as illustrated in Figure 4A. The top ten GO enrichments within each cluster were selected for subsequent analysis. The first cluster comprised terms such as “endoplasmic reticulum”, “endomembrane system”, and “carbohydrate transmembrane transport”, and demonstrated an initial increase in expression followed by a subsequent decrease (Figure 4B). The second cluster exhibited a general upward trend and included terms such as “cell proliferation”, “regulation of biological processes”, and “positive and negative regulation of cellular processes” (Figure 4C). The third cluster encompassed terms such as “myofibrils”, “contractile fibers”, and “actin cytoskeleton organization”, and displayed an overall declining trend (Figure 4D). The fourth cluster showed an initial decrease in expression followed by an increase and included GO terms such as “sterol metabolic processes”, “steroid metabolic processes”, and “lipid biosynthetic processes” (Figure 4E).

### 3.5. KEGG Enrichment Analysis of DEGs

**Figure 5:**
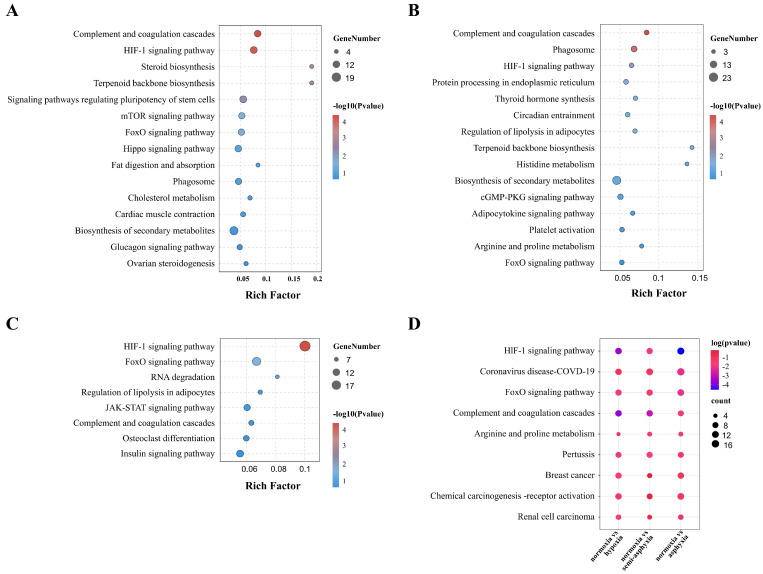
Bubble plots depict the KEGG enrichment analysis of differentially expressed genes across three comparative conditions: (**A**) normoxia vs. hypoxia, (**B**) normoxia vs. semi-asphyxia, and (**C**) normoxia vs. asphyxia. Additionally, matrix plots (**D**) illustrate the nine pathways that are commonly shared among these groups. Darker red indicates stronger statistical significance.

The bubble diagram delineates the signaling pathways enriched across the three comparison groups (Figure 5A–C, Table S9). Notably, the “HIF-1 signaling pathway” was identified among these pathways. Within the Metabolism subcategory, pathways such as “cholesterol metabolism,” “histidine metabolism,” “insulin metabolic pathway,” and “arginine and proline metabolism” are included. Furthermore, the “mTOR signaling pathway” and “JAK-STAT signaling pathway” are associated with signaling, while the “complement and coagulation cascades” and “phagosome” are linked to immune response. Pathways involved in cell function and regulation, such as “RNA degradation” and “regulation of stem cell pluripotency,” also demonstrated significant enrichment. The matrix diagram (Figure 5D) presents the nine pathways that were significantly enriched across all three comparison groups, including the HIF-1 signaling pathway, FoxO signaling pathway, and Complement and coagulation cascades, among others. The KEGG enrichment analysis of the 42 co-differentially expressed genes in Figure 2E revealed three significant pathways: “efferocytosis”, “butanoate metabolism”, and “arginine biosynthesis” (Table S10). These pathways involve three DEGs: *ARG1*, *SGK1*, and *aacs*. Additionally, *ARG1* is part of “arginine and proline metabolism,” and *SGK1* is involved in the “FoxO” and “mTOR” signaling pathways (Figure 5D).

### 3.6. Signaling Pathways Associated with Cell Homeostasis, Metabolism and Immunity

**Figure 6:**
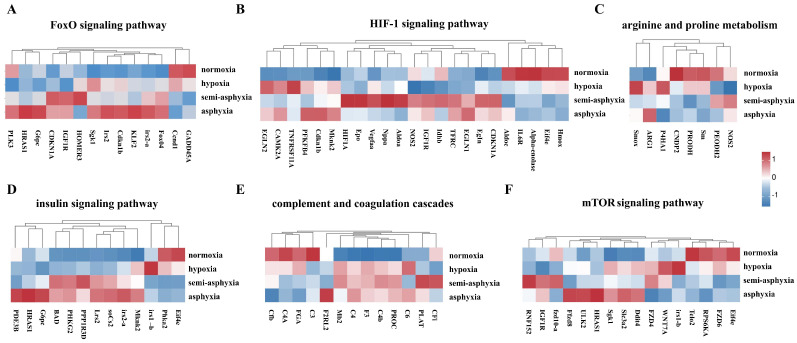
The heatmap depicts alterations in gene expression across six KEGG pathways. (**A**) FoxO signaling pathway, (**B**) HIF-1 signaling pathway, (**C**) arginine and proline metabolism, (**D**) insulin signaling pathway, (**E**) complement and coagulation cascades, and (**F**) mTOR signaling pathway. Blue denotes low expression levels, whereas red signifies high expression levels.

We employed a heatmap to visualize six KEGG pathways linked to the maintenance of cellular homeostasis, metabolic regulation, and immune modulation, namely the “FoxO signaling pathway”, “HIF-1 signaling pathway”, “mTOR signaling pathway”, “arginine and proline metabolism”, “complement and coagulation cascades”, and “insulin signaling pathway”. This visualization was used to depict the expression patterns of DEGs within these pathways (Figure 6). Analyzing the alterations in DEGs across these pathways provides insights into the physiological adaptations occurring in the liver of silver carp under hypoxic stress.

**Figure 7:**
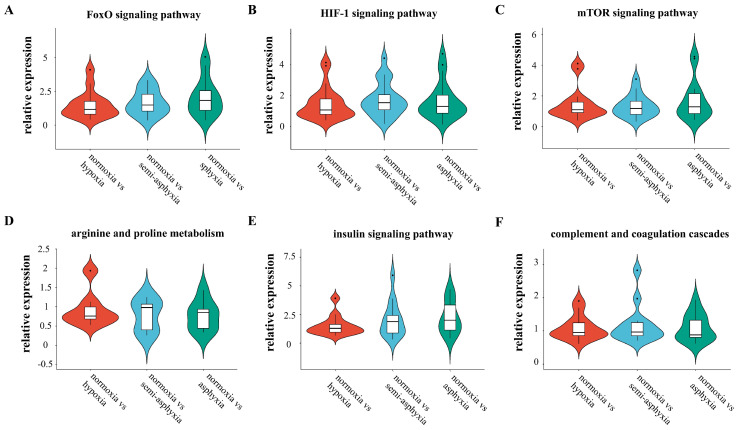
The violin plot illustrates the overarching trend of differential gene expression across six distinct pathways. (**A**) FoxO signaling pathway, (**B**) HIF-1 signaling pathway, (**C**) mTOR signaling pathway, (**D**) arginine and proline metabolism, (**E**) insulin signaling pathway, and (**F**) complement and coagulation cascades. In this type of plot, the width indicates the density of data points at a given value, whereas the height denotes the entire range of the data distribution.

The violin plot delineates the overarching expression patterns of differentially expressed genes across six distinct pathways (Figure 7). The findings indicate that with the escalation of hypoxic stress, the pathways maintain to cell homeostasis are progressively activated. Notably, the FoxO signaling pathway reaches its maximum activation in the asphyxia group, whereas the HIF-1 signaling pathway exhibits peak activity in the semi-asphyxia group. Metabolic pathways display bifurcated trends: as hypoxic stress intensifies, the arginine and proline metabolism pathway remains relatively stable, while the insulin signaling pathway undergoes significant activation. Regarding immune response, the complement and coagulation cascades demonstrate a trend of progressive suppression.

### 3.7. RT-qPCR Verification

**Figure 8:**
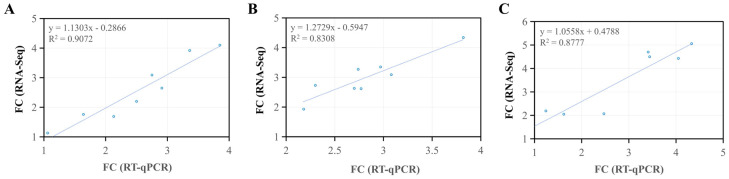
A scatter plot of Pearsons correlation analysis between the RT-qPCR data and RNA-Seq data. (**A**) normoxia vs. hypoxia, (**B**) normoxia vs. semi-asphyxia, and (**C**) normoxia vs. asphyxia. FC: fold change.

To validate the RNA-seq results, eight genes, including *EGLN3*, *SGK1*, *C3*, *IRS2*, *MKNK2*, *IRS1-b*, *HIF1α*, and *FOXO4*, were randomly selected for RT-qPCR. The amplification and melting curves for the genes were presented in Figure S2. The amplification curves were parallel in the exponential phase, suitable for qPCR analysis. All samples showed a single, sharp peak in the melt curve, indicating high specificity and reliable qPCR data. The results of statistical analysis demonstrated that the qPCR data were fully consistent with the RNA sequencing findings (Figure 8), and eight genes exhibited consistent upregulation trends across both detection methods (Table S11).

## 4. Discussion

In aquaculture systems, DO serves as a crucial environmental factor [24] that profoundly influences fish survival, growth, and reproduction [25]. Under conditions of hypoxic stress, fish adopt adaptive strategies, such as optimizing metabolic pathways, to decrease oxygen consumption while sustaining energy production, thereby maintaining physiological homeostasis [26]. Considering the liver’s essential function in substance metabolism and immune regulation [27], this study undertook a comparative transcriptomic analysis of silver carp liver tissues subjected to different levels of hypoxic stress. The aim was to elucidate the hepatic mechanisms underlying adaptation to hypoxia. The findings demonstrated a significant number of DEGs in the liver under varying levels of hypoxic stress (Figure 2). Utilizing KEGG pathway enrichment analysis, six significant pathways were identified, encompassing conditions from mild hypoxia to asphyxia. These included core hypoxia-adaption pathways crucial for maintaining cellular homeostasis, such as HIF-1, mTOR, and FoxO signaling pathway; pathways related to metabolism, notably arginine and proline metabolism as well as the insulin signaling pathway; and the complement and coagulation cascades, which play a role in immune regulation (Figure 6 and Figure 7).

### 4.1. Cellular Homeostasis Regulation Under Hypoxic Stress

The HIF-1 signaling pathway serves as a pivotal regulatory mechanism in the cellular response to hypoxia, significantly contributing to the maintenance of cellular homeostasis and adaptation to hypoxic environments [28]. Hypoxia-inducible factor-1 (HIF-1) is an essential transcription factor that becomes activated under hypoxic conditions, orchestrating the expression of numerous genes to enable cellular adaptation to low-oxygen conditions. Studies have demonstrated that HIF-1 sustains cellular homeostasis and facilitates adaptation to hypoxia through a variety of mechanisms, including the modulation of cellular metabolism, the promotion of angiogenesis, and the regulation of apoptosis [29,30,31]. Under hypoxic conditions, the expression of HIF-1α was significantly upregulated in liver of silver carp. This finding is consistent with previous research conducted in zebrafish, where HIF-1α expression remained stable and activated downstream target genes, including those involved in the regulation of the insulin-like growth factor 1 receptor (*IGF1R*) gene during early development [32]. Additionally, we observed a notable upregulation of *EGLN1* and *EGLN2*, which are essential regulators of HIF-1α stability. *EGLN* expression was up-regulated in rainbow trout (*Oncorhynchus mykiss*) liver tissue under hypoxic conditions, which is consistent with our results [33]. This observation supports earlier studies indicating that *EGLN* family proteins modulate HIF-1α stability through hydroxylation, thereby playing a crucial role in maintaining hepatic oxygen homeostasis [34]. Furthermore, *CAMK2A* exhibited significant upregulation, which plays a pivotal role in facilitating stress adaptation through calcium signaling pathways [35]. This concurrent upregulation suggests a strong protective response against hypoxic damage. Erythropoietin (*EPO*) is a crucial glycoprotein hormone that plays a vital role in sustaining tissue oxygenation by stimulating erythropoiesis in response to hypoxic environments [36]. Under conditions of hypoxic stress, the *EPO* gene exhibited marked upregulation within the semi-asphyxia group. This observation is potentially attributable to the interaction of HIF-1α with the enhancer region of the *EPO* gene, which subsequently activates transcription and enhances *EPO* synthesis under hypoxic conditions [37,38]. In addition, this phenomenon was also observed in zebrafish, where the *EPO* gene was significantly up-regulated at the Epo mRNA level in the liver after 3 h of hypoxia treatment [39].

The FoXO transcription factor family is involved in various cellular physiological processes, such as apoptosis, cell cycle regulation, and resistance to oxidative stress, through the modulation of gene expression. Empirical evidence indicates that, under conditions of acute stress, the FoxO signaling pathway plays a crucial role in the regulation of hepatic function and the organism’s stress response [40,41,42]. In the context of heat stress, the FoxO signaling pathway, in collaboration with mitochondrial-associated apoptotic pathways, prevents liver injury in tsinling lenok trout [43]. *SGK1* enhances cellular adaptation to hypoxic stress by promoting the phosphorylation of FoxO transcription factors, thereby regulating their nuclear activity and influencing cell metabolism and survival [44,45]. In our study, *SGK1* exhibited significant upregulation, highlighting its role as a pivotal kinase that facilitates cell survival and indicating a robust protective response against hypoxic damage [46]. In contrast, a downregulation was observed in DNA damage response genes, such as *GADD45A* and its homologs. The suppression of *GADD45A* may mitigate hypoxia-induced apoptotic stress, thereby enhancing cellular survival under low-oxygen conditions [47]. It has been found that the expression of *GADD45A* gene in rainbow trout liver tissues may gradually stabilize or show a decreasing trend under long-term hypoxic conditions, which may be related to the gradual adaptation of fish to the hypoxic environment and the initiation of other adaptive mechanisms [48]. Importantly, these regulatory mechanisms may be modulated by the duration of hypoxia and tissue specificity [49], offering a molecular framework for understanding interspecies variation in hypoxic responses among fish.

The mTOR signaling pathway is pivotal in modulating cellular growth, proliferation, and metabolism, especially under stress conditions such as hypoxia [50]. Research has demonstrated that the regulation of the mTOR pathway in hypoxic environments not only impacts cellular metabolic functions but is also intricately linked with autophagy, apoptosis, and cell differentiation processes [51,52]. In response to hypoxic stress, genes differentially expressed in the liver tissue of silver carp were significantly enriched within the mTOR signaling pathway, although the activation level was not markedly pronounced. This finding corroborates previous studies suggesting that hypoxia can activate the AMPK/REDD1 signaling axis to inhibit the mTOR pathway [53]. Within the mTOR pathway, only the genes *SGK1*, *IGF1R*, *DDIT4*, and *SLC3A2* were consistently upregulated under various degrees of hypoxic stress, with *SGK1* and IGF1R also participating in the FoxO and HIF-1 signaling pathways. *DDIT4*, a transcription factor induced by DNA damage, has been shown to regulate cell growth and survival by inhibiting mTOR signaling across different cell types. Under hypoxic conditions, *DDIT4* has been recognized as a critical intermediary connecting the HIF1α and mTOR signaling pathways, with its expression markedly elevated, as substantiated by several studies [54,55,56]. Additionally, the upregulation of *SLC3A2* gene expression within the mTOR signaling pathway under hypoxic stress is modulated by HIF1α, signifying a cellular adaptive response to environmental stressors.

**Figure 9:**
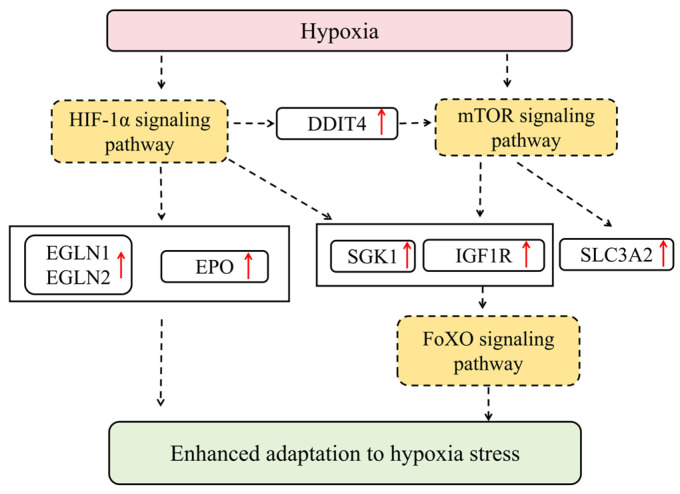
The molecular regulatory network of HIF-1, mTOR, and FoxO pathways in hypoxic silver carp liver. Red arrows represent gene upregulation.

In summary, under hypoxic stress, liver tissue enhances its ability to adapt to low oxygen conditions through a coordinated signaling network. *SGK1* inhibits FoxO transcription factors via phosphorylation, while the upregulation of *EGLN1* and *EGLN2* regulates the stability of HIF-1α. Concurrently, genes in the mTOR pathway—*SGK1*, *IGF1R*, *DDIT4*, and *SLC3A2*—remain consistently upregulated. Among these, *DDIT4* acts as a key mediator connecting HIF-1α and mTOR signaling. These changes collectively demonstrate how liver tissue integrates the FoxO, HIF-1α, and mTOR signaling axes to achieve adaptation to hypoxic environments (Figure 9).

### 4.2. Energy Metabolism Under Hypoxic Stress

The liver functions as a crucial organ for metabolic processes. Under hypoxic conditions, hepatocytes undergo a series of metabolic reprogramming events to adapt to oxygen-deficient environments, with significant alterations observed in arginine and proline metabolism [57]. In our study, the *ARG1* gene was consistently upregulated in the liver tissues of silver carp subjected to hypoxic stress. This finding is consistent with observations in colorectal cancer development, where cancer cells in hypoxic environments also exhibit significant upregulation of *ARG1* expression, which is closely associated with the reprogramming of arginine metabolism [58]. Additionally, studies have demonstrated that *PRODH* expression is upregulated in tumor hypoxic environments, promoting cellular autophagy through AMPK activation to enhance cell survival under low oxygen conditions [59,60]. In our study, we observed a consistent downregulation of *PRODH* in the liver tissues of silver carp subjected to hypoxic stress, which contrasts with observations made in tumor hypoxic microenvironments. This discrepancy indicates that the expression pattern of this gene may vary with the severity of hypoxic stress. Under hypoxic conditions, the reprogramming of arginine and proline metabolism facilitates cellular adaptation to low oxygen environments by modulating redox balance and energy metabolism [61].

The insulin signaling pathway represents a fundamental mechanism within the body for the regulation of blood glucose and lipid metabolism. Its principal role involves facilitating the uptake and utilization of glucose and fatty acids by tissue cells, thereby ensuring the homeostasis of glucose and lipid metabolism [62]. In our study, we observed a significant activation of the insulin signaling pathway in the liver tissues of silver carp subjected to hypoxic stress. This observation aligns with previous studies, suggesting that these alterations may be linked to the activation of the HIF. Notably, the inhibition of PHD enzymes can enhance hepatic insulin sensitivity through an HIF-2α-dependent mechanism, while concurrently diminishing glucagon sensitivity [63]. The influence of hypoxia on the hepatic insulin signaling pathway may also entail modifications in metabolic processes. Prior research has demonstrated that under hypoxic conditions, hepatic glucose and lipid metabolism undergo substantial changes, with the downregulation of the insulin signaling pathway being correlated with suppressed glycolysis/gluconeogenesis and enhanced fatty acid metabolism [64]. Under hypoxic conditions, the upregulation of *IRS2* within the insulin signaling pathway is particularly noteworthy. As a pivotal mediator of the insulin/IGF-1 signaling pathway [65], *IRS2* plays a critical role in regulating energy balance and glucose homeostasis in the central nervous system independently of leptin by inhibiting FoxO1 activity [66,67]. This modulation facilitates cellular metabolic adaptation and energy utilization. These findings underscore the importance of the FoXO signaling pathway not only in maintaining cellular homeostasis [68], but also in glucose metabolism. Additionally, our study identified a substantial upregulation of *PFKFB4*, a key glycolytic enzyme, whose increased expression augments glycolytic flux [69]. Consequently, under hypoxic stress, silver carp hepatocytes sustain blood glucose homeostasis through the regulation of various genes and signaling pathways, while concurrently undergoing significant modifications in glucose and lipid metabolism.

### 4.3. Immune Response Under Hypoxic Stress

The complement and coagulation cascade pathway is significantly modulated under conditions of hypoxic stress. This pathway is integral to immune responses, encompassing the activation of both the complement system and coagulation processes, which are involved in defense against foreign antigens and wound healing, respectively [70]. In our experiments, the genes *C3*, *PROC*, and *CFB* exhibited marked downregulation in response to acute hypoxic stress. In fish, complement component *C3* is a pivotal element of the complement cascade, and understanding its regulatory mechanisms is crucial for elucidating the immune response strategies of the fish [71]. Previous studies have demonstrated that *Aeromonas hydrophila* can suppress the complement pathway by degrading fish complement *C3*, thereby evading host immune defenses. This evasion strategy involves the secretion of metalloproteases by *A. hydrophila*, which effectively degrade *C3* in grass carp serum, resulting in the inhibition of the complement pathway and increased serum resistance of *A. hydrophila* [72]. This finding highlights the critical role of complement component *C3* in the immune defense of fish and elucidates the mechanisms by which pathogens evade immune responses by targeting the complement system. This indicates that *C3* serves a key regulatory function in immune responses subsequent to pathogen infection [73]. Additionally, studies have demonstrated that recombinant *C3* protein can mitigate post-infection inflammatory responses and histopathological damage, while enhancing the phagocytosis of *Streptococcus agalactiae* by monocytes and macrophages [74]. These results provide a theoretical framework for a more comprehensive understanding of the role of *C3* in fish immune defense.

We compared the differentially expressed genes between silver carp and crucian carp under hypoxic conditions, identifying several common KEGG pathways, such as the FoxO signaling pathway and arginine and proline metabolism. Notably, we also identified pathways uniquely in silver carp, including the HIF-1 signaling pathway and complement and coagulation cascades. These findings indicate that under hypoxic stress, the HIF-1 signaling pathway is more prominently activated in silver carp, and the immune response is more pronounced, suggesting a lower tolerance to hypoxia compared to crucian carp [75]. This study establishes a foundational framework for elucidating the response mechanisms of hypoxia-sensitive fish to hypoxic stress. However, it mainly relies on transcriptomic data and lacks the support of macro-phenotypic data directly associated with it. Therefore, future studies need to correlate and analyze the transcriptomic data with hard metrics such as survival performance, growth traits, and reproductive success of individuals, and verify which molecular markers can be stably inherited and co-selected with hypoxia-resistant phenotypes through multi-generation selection experiments.

## 5. Conclusions

In this study, a gradient hypoxic environment was established to examine the effects of hypoxia on the liver of silver carp through transcriptome analysis. Transcriptome sequencing of liver samples subjected to varying levels of hypoxia identified 628 DEGs. KEGG enrichment analysis further identified six pathways that were consistently and significantly enriched across all stress intensities, implicating maintenance of cellular homeostasis, metabolic regulation, and immune modulation. These findings reveal a complex molecular network underlying hepatic hypoxia adaptation in silver carp, enhancing our understanding of the molecular mechanisms involved. This research establishes a conceptual framework for interpreting piscine hypoxia tolerance and provides a theoretical basis for future breeding of hypoxia-resistant varieties.

## Figures and Tables

**Table 1:** RNA-seq statistics for silver carp liver under hypoxic conditions.

Group	Total Reads	Q20 (%)	Q30 (%)	GC (%)	Mapped Reads	Mapping Rate
normoxia_1	34,766,763	98.47	95.27	46.38	28,057,470	80.71%
normoxia_2	31,415,454	98.6	95.63	47.53	25,824,886	82.21%
normoxia_3	25,187,609	98.47	95.26	46.75	19,952,463	79.21%
hypoxia_1	21,492,620	98.74	96.02	46.94	16,444,772	76.51%
hypoxia_2	21,471,726	98.67	95.8	46.69	17,470,611	81.37%
hypoxia_3	30,473,909	98.52	95.41	46.25	24,092,095	79.05%
semi-asphyxia_1	24,130,833	98.8	95.99	46.3	19,713,236	81.69%
semi-asphyxia_2	20,237,307	98.75	96.03	47.53	16,277,372	80.43%
semi-asphyxia_3	38,993,725	98.64	95.97	45.42	21,903,041	56.17%
asphyxia_1	22,288,275	98.81	96.24	46.85	18,263,126	81.94%
asphyxia_2	24,868,992	98.79	96.18	46.49	19,252,710	77.42%
asphyxia_3	25,523,344	98.73	95.97	47.02	20,345,683	79.71%

## Data Availability

The data presented in this study are available in the article. Raw transcriptome sequencing data are available at the NCBI SRA database (Accession numbers: SRR18863890–SRR18863901).

## Supplementary Materials

The following supporting information can be downloaded at: https://www.mdpi.com/article/10.3390/ani15243577/s1, Figure S1. Principal component analysis (PCA) plots showing repeatability and heterogeneity of sequencing samples. Figure S2. The amplification and melting curves of the genes analyzed by RT-qPCR. Table S1. The DO concentrations of each experimental group in this and previous study. Table S2. Primers used for RT-qPCR; Table S3. Number of expressed genes detected in samples from each group; Table S4: The FPKM values for genes across each sample; Table S5: Differentially expressed genes identified from pairwise comparisons; Table S6: The distribution of differentially expressed genes in Venn diagram; Table S7: Expression profiles of 42 shared DEGs; Table S8: GO enrichment analysis of differentially expressed genes; Table S9. KEGG enrichment analysis of differentially expressed genes; Table S10. The KEGG enrichment analysis of the 42 overlap genes among “normoxia vs. hypoxia”, “normoxia vs semi-asphyxia” and “normoxia vs asphyxia”. Table S11. Comparisons of RNA-seq and RT-qPCR results.
