# Supplementary material for: Elucidating the Molecular Network Underpinning Hypoxia Adaptation in the Liver of Silver Carp (Hypophthalmichthys molitrix) via Transcriptome Analysis
Source: Animals (Basel). 2025 Dec 12;15(24):3577. doi: 10.3390/ani15243577 (PMC12729696; doi:10.3390/ani15243577)
Supplement: Supplementary file 1 [file animals-15-03577-s001.zip › Table S1.pdf]

Table S1. The DO concentrations of each experimental group in this and previous study.

	**hypoxia group**	**semi-asphyxia group**	**asphyxia group**
DO in this study	0.75 ± 0.05 mg/L	0.60 ± 0.04 mg/L	0.27 ± 0.03 mg/L
DO in References [13]	0.75 ± 0.04 mg/L	0.58 ± 0.06 mg/L	0.27 ± 0.06 mg/L
DO in References [19]	0.76 ± 0.03 mg/L	0.62 ± 0.09 mg/L	0.27 ± 0.06 mg/L

References

[13] Li, X.; Ling, C.; Wang, Q.; Feng, C.; Luo, X.; Sha, H.; He, G.; Zou, G.; Liang, H. Hypoxia stress induces tissue damage , immune defense , and oxygen transport change in gill of silver carp (*Hypophthalmichthys molitrix*): evaluation on hypoxia by using transcriptomics. *Front Mar Sci* **2022**, *9*, p. 15.

[19] Feng, C.; Li, X.; Sha, H.; Luo, X.; Zou, G.; Liang, H. Comparative transcriptome analysis provides novel insights into the molecular mechanism of the silver carp (*Hypophthalmichthys molitrix*) brain in response to hypoxia stress. *Comp Biochem Physiol Part D Genomics Proteomics* **2022**, *41*, p. 100951.
