# Supplementary material for: Elucidating the Molecular Network Underpinning Hypoxia Adaptation in the Liver of Silver Carp (Hypophthalmichthys molitrix) via Transcriptome Analysis
Source: Animals (Basel). 2025 Dec 12;15(24):3577. doi: 10.3390/ani15243577 (PMC12729696; doi:10.3390/ani15243577)
Supplement: Supplementary file 1 [file animals-15-03577-s001.zip › Table S11.pdf]

Table S11. Comparisons of RNA-seq and RT-qPCR results.

Gene abbreviation	Gene description	Fold-change (normoxia vs hypoxia)		Fold-change (normoxia vs semi-asphyxia)		Fold-change (normoxia vs asphyxia)	
		qPCR	RNA-seq	qPCR	RNA-seq	qPCR	RNA-seq
EGLN3	Egl nine homolog 3	1.64	1.76	2.70	2.63	2.47	2.07
SGK1	Serum and glucocorticoid-regulated kinase 1	3.85	4.10	3.08	3.09	4.05	4.43
C3	Complement component C3	2.50	2.20	3.82	4.34	3.44	4.5
IRS2	Insulin receptor substrate 2	2.75	3.09	2.30	2.73	4.33	5.06
mknk2	MAP kinase-interacting serine/threonine-protein kinase 2	3.36	3.92	2.97	3.35	*3.41*	4.7
irs1-b	Insulin receptor substrate 1-B	2.91	2.65	2.77	2.62	1.62	2.05
HIF1A	Hypoxia-inducible factor 1-alpha	1.06	1.13	2.74	3.27	0.94	1.53
Foxo4	Forkhead box protein O4	2.13	1.69	2.18	1.93	1.24	2.19
