# Supplementary material for: Elucidating the Molecular Network Underpinning Hypoxia Adaptation in the Liver of Silver Carp (Hypophthalmichthys molitrix) via Transcriptome Analysis
Source: Animals (Basel). 2025 Dec 12;15(24):3577. doi: 10.3390/ani15243577 (PMC12729696; doi:10.3390/ani15243577)
Supplement: Supplementary file 1 [file animals-15-03577-s001.zip › Table S2.pdf]

Table S2. Primers used for RT-qPCR.

Primer names	Primer sequences (5′–3′)
irs2-F	CGTCTCAGTTGTTCCCGTCA
irs2-R	CAAGTGTCTGTGTTGCCGTG
C3-F	CCACACCTGTCAGATGGACC
C3-R	CTTCTCAACAGGTCTGGCGT
egln3-F	GGACACGCAGTTGGAGACTT
egln3-R	CCGTCGTTGAGAATCCCACA
mknk2-F	GCCCATAGAGACCTGAAGCC
mknk2-R	GAACGCTTCCACAACTTCCG
SGK1-F	CGAACCAAGAGACTGGGCTT
SGK1-R	CCCGTCACGTTAGGGTTGAA
HIF1A-F	GACTTGGACAGTCTCGCTCC
HIF1A-R	CCCTCCGAATTTTGCAACCG
Foxo4-F	ACTTCCACAGCTGGCCTAAC
Foxo4-R	GTTGGTGAAACCATCGCCAC
IRS1-b-F	TCTGCTGGAAGCCATGAAGG
IRS1-b-R	TTCTGTCCGTGGACGTCTTG
40s-F	CGCAACAACGGCAAGAAACT
40s-R	ACACGTCAACAGCCTGTCTC
