# Supplementary material for: Elucidating the Molecular Network Underpinning Hypoxia Adaptation in the Liver of Silver Carp (Hypophthalmichthys molitrix) via Transcriptome Analysis
Source: Animals (Basel). 2025 Dec 12;15(24):3577. doi: 10.3390/ani15243577 (PMC12729696; doi:10.3390/ani15243577)
Supplement: Supplementary file 1 [file animals-15-03577-s001.zip › Table S3.pdf]

Table S3. Number of expressed genes detected in samples from each group.

Sample	Expressed Gene	Total Gene
normoxia_1	20,654	30,073
normoxia_2	20,537	30,073
normoxia_3	19,820	30,073
hypoxia_1	20,020	30,073
hypoxia_2	19,543	30,073
hypoxia_3	19,710	30,073
semi-asphyxia_1	19,508	30,073
semi-asphyxia_2	19,618	30,073
semi-asphyxia_3	20,570	30,073
asphyxia_1	19,627	30,073
asphyxia_2	19,881	30,073
asphyxia_3	19,901	30,073
