# Supplementary figures and images for: Elucidating the Molecular Network Underpinning Hypoxia Adaptation in the Liver of Silver Carp (Hypophthalmichthys molitrix) via Transcriptome Analysis
Source: Animals (Basel). 2025 Dec 12;15(24):3577. doi: 10.3390/ani15243577 (PMC12729696; doi:10.3390/ani15243577)

**Figure S2.** The amplification and melting curves of the genes analyzed by RT-qPCR.

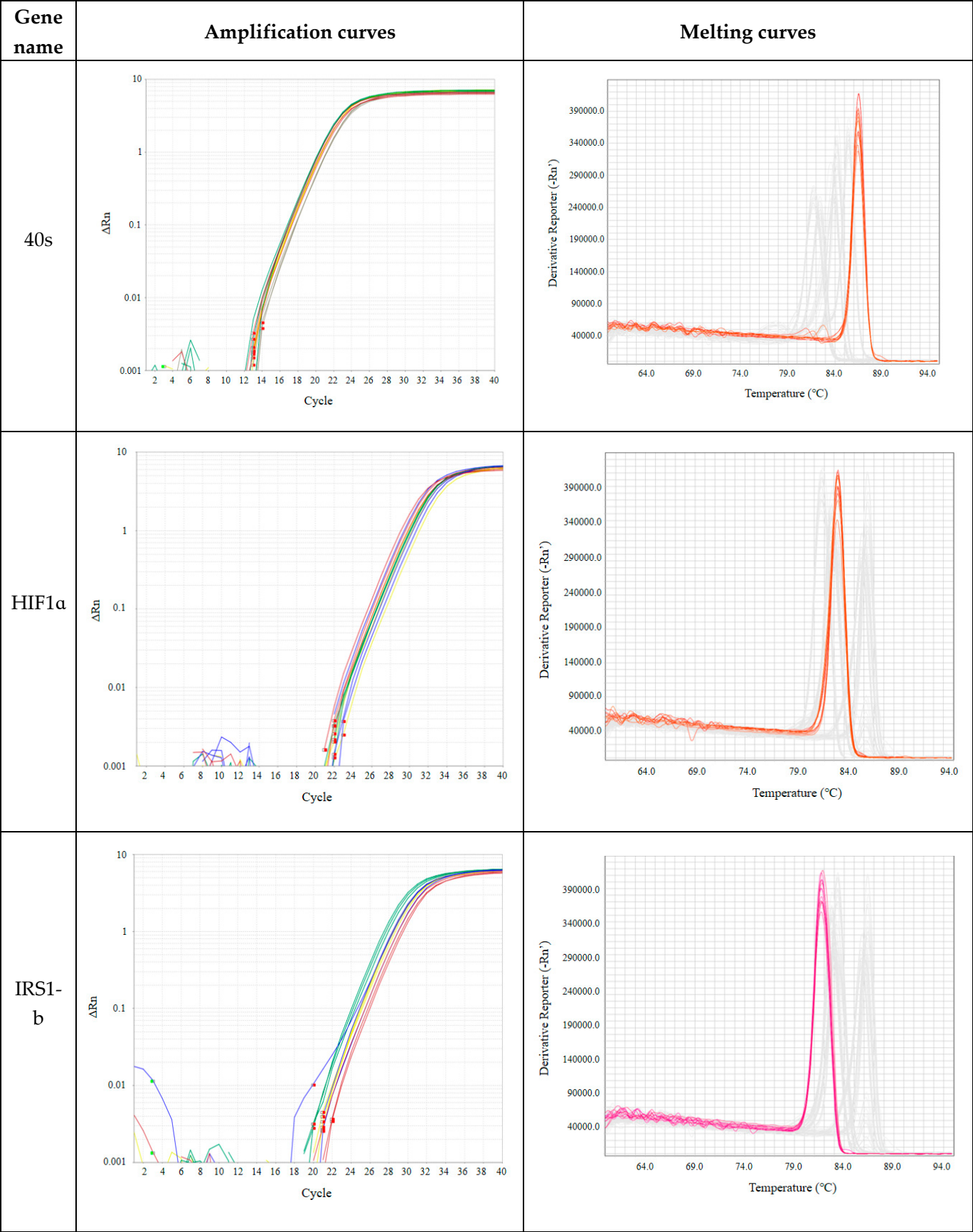

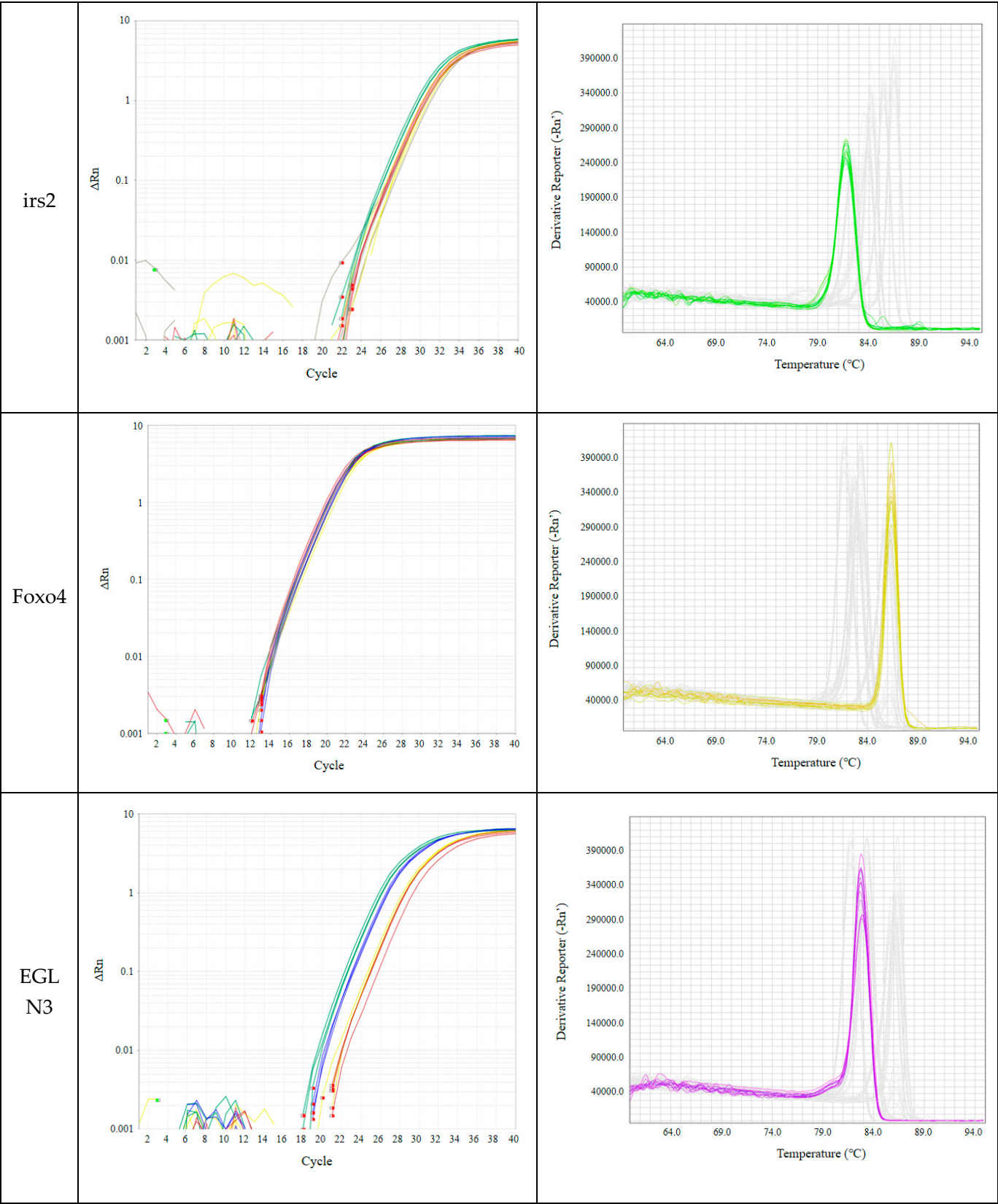

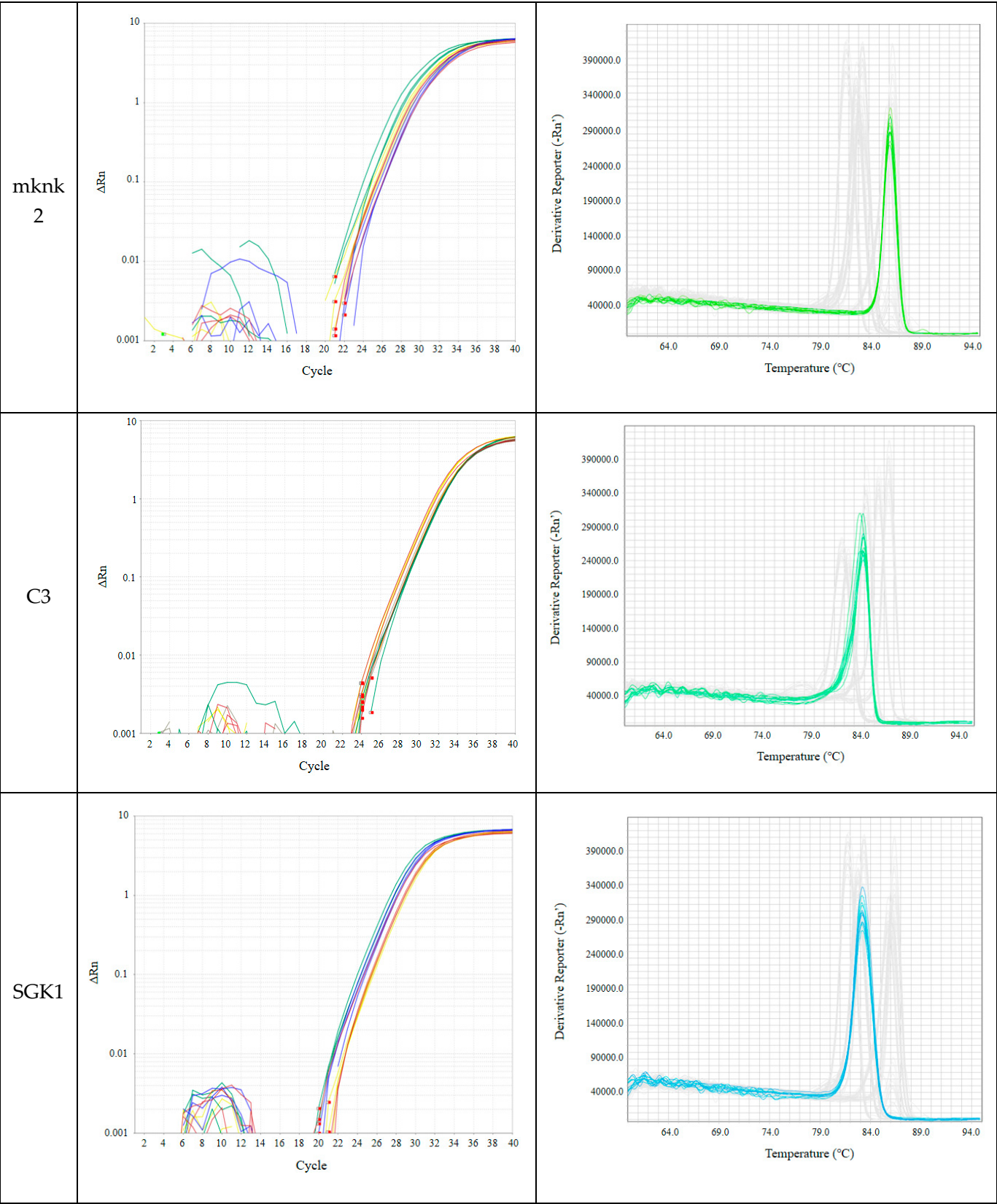

Supplement: Supplementary file 1 [file animals-15-03577-s001.zip › Figure S2.pdf]
